# Breast cancer risk, worry, and anxiety: Effect on patient perceptions of false-positive screening results

**DOI:** 10.1016/j.breast.2020.02.004

**Published:** 2020-02-13

**Authors:** Janie M. Lee, Kathryn P. Lowry, Jessica E. Cott Chubiz, J. Shannon Swan, Tina Motazedi, Elkan F. Halpern, Anna N.A. Tosteson, G. Scott Gazelle, Karen Donelan

**Affiliations:** a Department of Radiology, University of Washington, Seattle Cancer Care Alliance, Seattle, WA, USA; b Washington University School of Medicine, St. Louis, MO, USA; c Institute for Technology Assessment, Department of Radiology, Massachusetts General Hospital, Boston, MA, USA; d Department of Medicine, Baylor College of Medicine, Houston, TX, USA; e The Dartmouth Institute for Health Policy and Clinical Practice and Norris Cotton Cancer Center, Geisel School of Medicine at Dartmouth, Lebanon, NH, USA; f Mongan Institute Health Policy Center, Massachusetts General Hospital, Boston, MA, USA

## Abstract

**Objective:**

The impact of mammography screening recall on quality-of-life (QOL) has been studied in women at average risk for breast cancer, but it is unknown whether these effects differ by breast cancer risk level. We used a vignette-based survey to evaluate how women across the spectrum of breast cancer risk perceive the experience of screening recall.

**Methods:**

Women participating in mammography or breast MRI screening were recruited to complete a vignette-based survey. Using a numerical rating scale (0–100), women rated QOL for hypothetical scenarios of screening recall, both before and after benign results were known. Lifetime breast cancer risk was calculated using Gail and BRCAPRO risk models. Risk perception, trait anxiety, and breast cancer worry were assessed using validated instruments.

**Results:**

The final study cohort included 162 women at low (n = 43, 26%), intermediate (n = 66, 41%), and high-risk (n = 53, 33%). Actual breast cancer risk was not a predictor of QOL for any of the presented scenarios. Across all risk levels, QOL ratings were significantly lower for the period during diagnostic uncertainty compared to after benign results were known (p < 0.05). In multivariable regression analyses, breast cancer worry was a significant predictor of decreased QoL for all screening scenarios while awaiting results, including scenarios with non-invasive imaging alone or with biopsy. High trait anxiety and family history predicted lower QOL scores after receipt of benign test results (p < 0.05).

**Conclusions:**

Women with high trait anxiety and family history may particularly benefit from discussions about the risk of recall when choosing a screening regimen.

## Highlights

• Impact of screening recall on quality-of-life does not vary by breast cancer risk.
• Breast cancer worry predicts lower quality-of-life ratings while awaiting results.
• Quality-of-life ratings improve after receipt of benign results.
• High trait anxiety predicts lower quality-of-life after benign results are known.

## 1 Introduction

Mammography is the current standard for breast cancer screening as it has been shown to decrease breast cancer mortality in the general population [1,2]. However, in women at high risk for breast cancer such as BRCA mutation carriers, mammography detects less than half of all breast cancers, possibly due to young patient age and increased breast density, or tumor pathologic factors [3,4]. As such, women at high risk for breast cancer are offered more intensive screening at younger ages and with supplemental modalities such as magnetic resonance imaging (MRI) [5,6] which has been shown to improve cancer detection [7,8] and reduce the incidence of advanced stage breast cancers [9] when used for high-risk screening.

Although there are benefits to more intensive screening regimens in high-risk women, an inherent drawback to screening at younger ages and with multiple modalities is the increased frequency of recall for additional imaging and interventions in women who do not have cancer [10]. Studies that have examined the impact of false positive screening mammograms in average risk women have suggested that the consequences of screening recalls include increased worry about breast cancer, psychological distress, and anxiety [[11], [12], [13], [14], [15], [16]]. The majority of these studies have suggested that these effects are transient, although some studies suggest that even these short-term effects may affect health behaviors such as future screening decisions [17]. Despite these findings, a survey of US women’s attitudes toward false-positive mammography results found that women largely view false-positive test results as an acceptable consequence of screening [18].

While the impact of false positive examinations has been previously studied in average risk women, it is not known how these perceptions compare to those of women who are at high risk for breast cancer. As additional genetic and other risk factors for breast cancer are identified that qualify women for high-risk screening, it is important to understand the experience of false-positive screening examinations in this patient population to inform clinical guidelines and facilitate shared decision-making. In addition, this information is needed to inform cost-effectiveness analyses of high-risk screening strategies, as the cost-effectiveness of screening mammography has been shown to be highly sensitive to small, short-term effects on quality-of-life (QOL) related to the screening test [19].

The purpose of this study was to better understand how women across a spectrum of breast cancer risk perceive the impact of false-positive screening examinations and biopsies on QOL. We hypothesized that perceived QOL during and after the experience of screening recall would vary by underlying breast cancer risk.

## 2 Methods

This study was HIPAA-compliant and approved by the Institutional Review Board of Massachusetts General Hospital.

### 2.1 Subject recruitment

We recruited women ages ≥25 scheduled for routine mammographic screening or high-risk breast MRI screening at Massachusetts General Hospital from July 2011 through December 2012. Women were eligible to participate regardless of history of prior screening recall and/or biopsy, or history of prior breast cancer. Eligible women received a written invitation by mail to participate in the study four weeks prior to their scheduled exam and were subsequently contacted by telephone after 10 days if no response was received. Participants were given the option to complete the survey online, by mail, or with assistance over the telephone with a study coordinator. Women could complete the survey at any time during the four weeks leading up to their screening exam appointment. On the day of the screening exam, the survey was closed.

### 2.2 Survey instrument

We developed a vignette-based survey to assess women’s beliefs about breast cancer risk and the experience and consequences of false positive screening examinations leading to additional imaging and procedures. The survey instrument comprised of a series of vignettes describing temporary health state scenarios related to recall from screening mammography or MRI which participants were asked to rate using a numerical QOL rating scale[20], as detailed below. Demographic information was also collected for each participant, including age, sex, and race/ethnicity, personal breast cancer screening history (including prior screening recalls or biopsies), prior breast cancer history, and breast cancer risk factors. Previously validated measures of breast cancer worry, trait anxiety, and numeracy were also administered (described below). The survey was available in paper format and online, using the online REDCap survey design tool (version 5.0.15, Vanderbilt University). The complete version of the survey is available in the online Supplement.

### 2.3 Vignettes for false positive screening recall

Participants were presented with a series of vignettes describing the temporary health states related to recall after a) abnormal screening mammography; or b) abnormal screening breast MRI. Descriptions of temporary health states such as vignettes can be used to elicit preferences and quality-of-life perceptions from the general population[21,22], and have been previously used to quantify utilities for early and advanced breast cancer stages [23,24] and breast cancer treatment effects [24]. For this study, vignettes were developed by a team of investigators with expertise in breast imaging and image-guided procedures and survey science. For each vignette, participants were asked to rate the experience after recall for a scenario requiring additional non-invasive imaging alone, and a scenario in which a biopsy was performed. All vignettes included communication of benign results after non-invasive diagnostic imaging, or after a percutaneous biopsy was performed. For each scenario, we asked participants to rate QOL as defined by health utility theory using a 0 to 100 numerical scale (anchored by 0 = death, 100 = optimal health)[20], both during the period of diagnostic uncertainty and after benign results were known. The survey was pilot tested with 29 participants meeting study eligibility criteria, and vignette text was revised for clarity based on pilot participant feedback prior to the launch of the full study.

### 2.4 Actual and perceived breast cancer risk

Demographic information and breast cancer risk factors were used to calculate lifetime breast cancer risk using the Gail model [25] and the BRCAPRO [26] risk model in women without and with a family history of breast cancer, respectively. Each individual’s breast cancer risk was then stratified into one of the following risk groups: *low/average risk* (lifetime risk <15%); *intermediate risk* (lifetime risk 15–20%); and *high risk* (lifetime risk >20%) [5].

To assess perceived breast cancer risk, participants were asked to estimate their risk of developing breast cancer in their lifetime (expressed as a percentage, from 0 to 100%). Women who endorsed a personal history of breast cancer were asked to estimate their risk of developing a new or recurrent breast cancer in their lifetime (expressed as a percentage, from 0 to 100%).

### 2.5 Trait anxiety

The Spielberger State Trait Anxiety Index (STAI) is an established instrument used to assess general and trait anxiety [27]. For the current study, participants completed the trait-anxiety component of the instrument, which assigns a percentile for each participant based on her index score and age. Anxiety percentiles in the highest quartile (≥75%) were considered *high anxiety*.

### 2.6 Numeracy

Numeracy was assessed using the following multiple choice items taken from a previously validated instrument[28]: 1) Which of the following numbers represents the biggest risk of getting a disease? (Options: 1 in 100, 1 in 1000, 1 in 10, or Don’t Know/Not Sure); and 2) Which of the following represents the biggest risk of getting a disease (Options: 1%, 10%, 5%, or Don’t Know/Not Sure). Women who answered both items correctly were considered to have high numeracy.

### 2.7 Breast cancer worry

The Lerman Breast Cancer Worry Scale [29] is a 3-item scale which assesses the impact of concern about breast cancer on daily function and activities. We assessed breast cancer worry by adapting two of the items from the scale: “How often do you worry about developing a new or recurrent breast cancer?” and “How much does worrying about a new or recurrent breast cancer interfere with your daily life?” Women rated both questions on a scale of 1 (not at all) to 7 (all the time). Responses of 6 or 7 were considered to indicate “high” breast cancer worry.

### 2.8 Regression and statistical analysis

We performed t-tests, chi-square tests, univariate and multivariable regression to examine whether short-term quality of life ratings for each vignette were associated with the following variables: age (continuous), race, numeracy, personal and family history of breast cancer, breast cancer risk category (low/intermediate versus high), breast cancer worry and trait anxiety. Because breast cancer risk perception has been previously shown to be inaccurate among women[30,31], we performed univariate regression to examine the relationship between perceived risk and QOL ratings in the vignette. Spearman’s correlation was used to compare perceived risk by actual risk category. All statistical analyses were performed in SAS (version 9.4, Cary, NC), using stepwise linear regression.

## 3 Results

### 3.1 Participant recruitment and characteristics

**Fig. 1:**
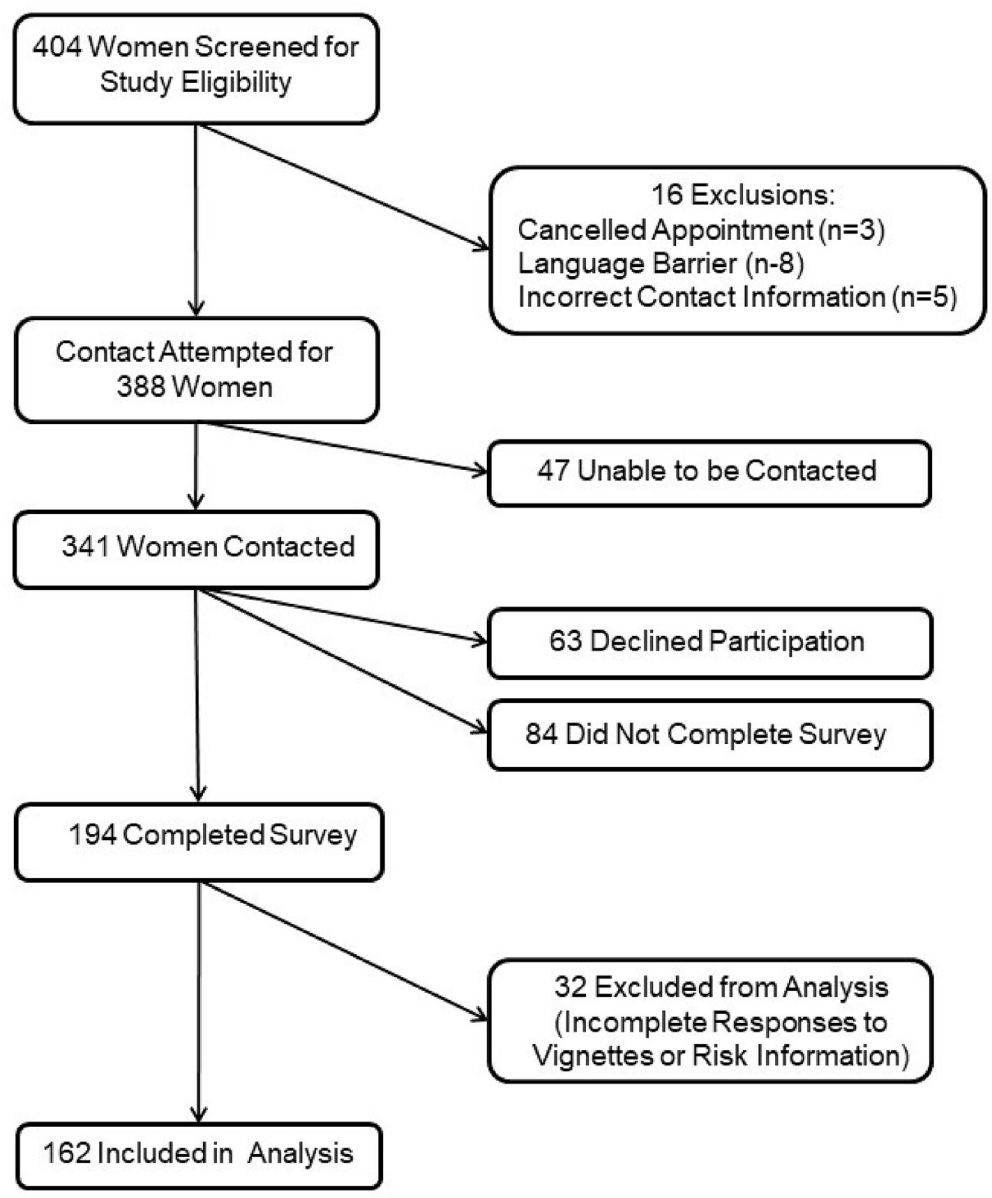
Survey recruitment and participation. Contact rate was 341/404 (84%), cooperation rate was 194/341 contacted women (57%), and response rate was 194/388 eligible women (50%).

Participant recruitment is summarized in Fig. 1. Of the 404 women who were initially identified, 16 women were excluded due to: incorrect contact information (n = 5), language barrier (n = 8), or cancelled screening exam (n = 3). An additional 47 were unable to be reached via mail or phone. Of 341 women who were successfully contacted, 194 completed the survey (cooperation rate: 194 of 341 women who were contacted, 57%; response rate: 194 of 388 women who were eligible, 50%). Of the 147 women who did not complete the survey, 63 (43%) refused the survey, 56 (38%) did not log on to the online survey, and 28 (19%) logged on but did not complete any survey items. Finally, we excluded women with incomplete responses for screening scenario questions or incomplete information for risk calculation (32 women).

The cohort included for final analysis included 162 women at low (n = 43, 26%), intermediate (n = 66, 41%) and high risk (n = 53, 33%). Characteristics of the final cohort are summarized in Table 1. Of note, 95% of the women surveyed were non-Hispanic white, and more than two-thirds had a college degree or higher. Approximately 43% of the women had a personal history of breast cancer, and approximately half had a family history of breast cancer. Almost all women (99%) had previously undergone mammography for breast cancer screening. Approximately 74% of women had undergone a biopsy in the past, and 59% had experienced a prior benign biopsy due to a false-positive screen. STAI trait anxiety scores were evenly distributed across all four quartiles. Ten percent of participants indicated “high worry” about breast cancer.

**Table 1:** Characteristics of women completing vignettes based on survey responses (n = 162).

	Completed vignettes, n (%)
Age, mean (range)	53 (26–85)
Race/Ethnicity (missing: n = 2)	
White, non-Hispanic	154 (95)
White, Hispanic	1 (1)
Nonwhite, non-Hispanic	4 (3)
Nonwhite, Hispanic	1 (1)
Education level (missing: n = 2)	
College graduate or higher	109 (67)
Numeracy Questions (missing: n = 3)	
Both correct	114 (70)
One correct	19 (12)
None correct	26 (16)
Personal history of breast cancer	70 (43)
Invasive carcinoma	41 (59)
Ductal carcinoma in situ	19 (27)
Unknown type	10 (14)
First degree family history of breast cancer	80 (49)
Screening History	
Prior screening mammogram (missing: n = 1)	160 (99)
Prior breast MRI (missing: n = 1)	122 (75)
Prior screening recall (missing: n = 4)	117 (72)
Prior biopsy (missing = 2)	119 (74)
Lifetime breast cancer risk∗	
Low/Average (<15%)	43 (27)
Intermediate (15–20%)	66 (41)
Personal history of breast cancer	56 (35)
Personal history of lobular carcinoma in situ (LCIS)	10 (6)
High (>20%)	53 (33)
Known germline mutation	41 (25)
History of chest radiation	9 (6)
Untested 1P^st^P degree relative of germline mutation carrier	2 (1)
Lifetime risk >20% based on risk models	1 (1)
Perceived lifetime breast cancer risk (%), mean (range) (missing: n = 15)	51% (1–100%)
Trait Anxiety (STAI)	
Top Quartile	45 (28)
51-75P^th^P percentile scores	45 (28)
26-50P^th^P percentile scores	35 (22)
Bottom Quartile	37 (23)

^a^Estimated using the Gail and BRCAPRO models in women without and with family history of breast cancer, respectively.

### 3.2 Perceived versus actual breast cancer risk

**Fig. 2:**
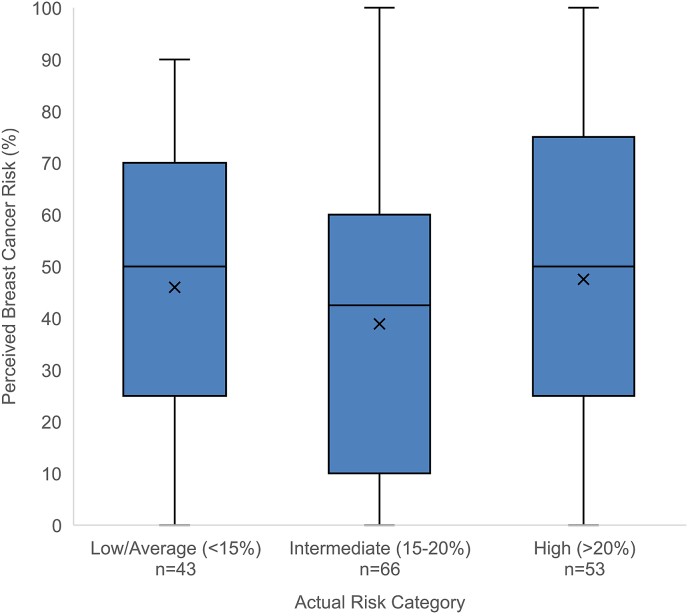
Perceived versus actual lifetime risk of breast cancer. Perceived risk of breast cancer was estimated as a percentage by study participants; actual risk was calculated using the Gail and BRCAPRO models in women without and with family history of breast cancer, respectively. Perceived risk did not vary by actual breast cancer risk category (low/average < 15%, intermediate 15–20%, versus high >20%). Means for each category are denoted by ‘X’.

The majority of women in the low/average and intermediate breast cancer risk groups overestimated their breast cancer risk (81% of low/average risk women and 68% of intermediate risk women). Perceived risk of breast cancer did not vary by actual risk category (Spearman’s r = 0.048, p = 0.54); average perceived lifetime risk of breast cancer was 46%, 39%, and 48% in low/average, intermediate, and high-risk women, respectively (Fig. 2). Among low/average risk women, 75% incorrectly reported that they were at high risk with >20% lifetime risk of breast cancer.

### 3.3 QOL ratings during and after diagnostic uncertainty

Univariate and multivariable regression results for QOL ratings during screening mammography recall are shown in Table 2 (QOL scores during recall for additional non-invasive imaging) and Table 3 (QOL scores during recall with biopsy performed). Additional tables are included in the online appendix with QOL scores during recall from screening MRI for additional non-invasive imaging (Table E1) and with MR-guided biopsy performed (Table E2). Results from regression models using stepwise selection are shown; results using forward and backward selection produced similar results (not shown).

**Table 2:** Quality-of-life (QOL) scores during and after recall from screening mammography for non-invasive additional imaging.

Characteristic	During Diagnostic Uncertainty after Mammography Recall			After Benign Results Received after Mammography Recall		
	Mean QOL Score (SD)	Univariate p value	Multivariable p value	Mean QOL Score (SD)	Univariate p value	Multivariable p value
Age	54.5 (26.2)	0.51		67.2 (25.8)	0.70	0.08
Race		0.21			0.40	
White	54.3 (26.2)			67.2 (25.8)		
Nonwhite	60.1 (27.0)			77.0 (24.9)		
Numeracy Questions		**0.02**	0.11		0.39	
Both correct	57.8 (25.7)			66.0 (26.1)		
1+ incorrect	46.9 (26.3)			69.9 (25.2)		
Family History of BC		0.42			0.51	**0.04**
Yes	56.2 (26.6)			65.8 (26.3)		
No	52.9 (25.9)			68.5 (25.4)		
Personal History of BC		0.73			0.80	
Yes	52.3 (30.6)			66.8 (28.0)		
No	54.1 (23.4)			68.1 (23.3)		
Actual BC Risk		0.10			0.43	
Low/Intermediate	52.2 (26.9)			66.0 (24.8)		
High	59.4 (24.4)			69.5 (27.9)		
Perceived BC Risk (%)	54.9 (26.7)	0.29		66.3 (26.2)	0.46	
Anxiety Level		0.27			**0.03**	**0.04**
Top quartile	50.8 (27.9)			60.1 (27.5)		
Bottom 3 quartiles	56.0 (25.6)			69.9 (24.7)		
BC worry^a^		**<0.001**	**<0.001**		0.35	
High worry	32.8 (28.5)			61.6 (31.9)		
Low-moderate worry	57.1 (24.8)			67.8 (25.0)		

BC=Breast Cancer.

a High worry was defined as a score ≥6 on the Breast Cancer Worry scale.

**Table 3:** Quality-of-life (QOL) scores during and after recall from screening mammography with biopsy performed.

Characteristic	During Diagnostic Uncertainty After Biopsy			After Benign Results Received After Biopsy		
	Mean QOL Score (SD)	Univariate p value	Multivariable p value	Mean QOL Score (SD)	Univariate p value	Multivariable p value
Age	45.4 (26.9)	0.45		67.2 (27.0)	0.48	0.12
Race		0.13			0.52	
White	44.9 (26.8)			67.0 (27.2)		
Nonwhite	63.8 (31.7)			75.0 (25.0)		
Numeracy Questions		0.06	0.11		0.53	
Both correct	48.0 (26.7)			66.3 (27.2)		
1+ incorrect	39.3 (26.7)			69.2 (26.6)		
Family History of BC		0.81			**0.05**	**0.04**
Yes	45.1 (27.1)			63.1 (28.9)		
No	45.7 (26.9)			71.2 (24.5)		
Personal History of BC		0.70			0.92	
Yes	44.0 (30.0)			67.5 (28.6)		
No	46.0 (25.0)			66.9 (25.9)		
Risk		0.30			0.50	
Low/Intermediate	43.9 (26.8)			66.2 (26.6)		
High	48.6 (27.1)			69.2 (27.9)		
Perceived BC Risk (%)	45.3 (27.0)	0.37		66.1 (26.9)	0.18	
Anxiety Level		0.35			**0.04**	**0.02**
Top quartile	42.2 (25.8)			60.0 (27.6)		
Bottom 3 quartiles	46.7 (27.3)			69.9 (26.3)		
BC worry^a^		**0.001**	**0.002**		0.18	
High worry	25.8 (25.6)			58.9 (31.8)		
Low-moderate worry	47.7 (26.2)			68.1 (26.3)		

BC=Breast Cancer.

a High worry was defined as a score ≥6 on the Breast Cancer Worry scale.

**Fig. 3:**
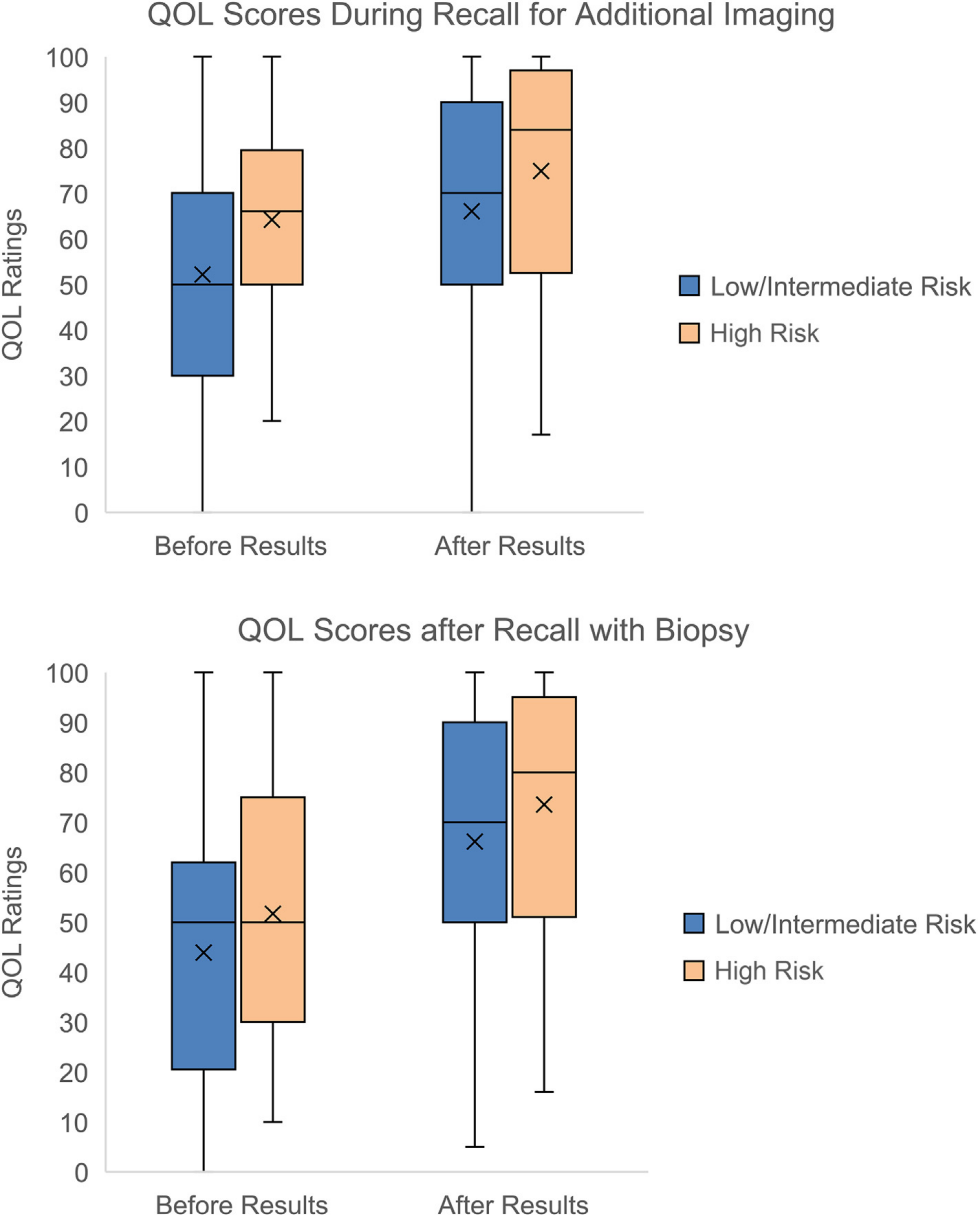
A–3B. QOL ratings by participants across risk categories (low/intermediate versus high) for scenarios of screening mammography recall with additional imaging (panel A) and biopsy (panel B). In both scenarios, QOL ratings significantly increased after receipt of benign results compared to before results were known (p < 0.05 for all scenarios). QOL ratings for each scenario did not differ by breast cancer risk category. Blue bars = low/intermediate risk women; orange bars = high-risk women. Means for each category are denoted by ‘X’.

QOL ratings did not vary by risk group for any of the presented scenarios across breast cancer risk groups (Fig. 3). Across all risk levels and all screening scenarios, QOL scores were significantly lower during the period of diagnostic uncertainty versus after benign results were received; on average, QOL scores increased by 16 after receipt of benign results compared to the period of uncertainty (p < 0.05 for all scenarios). In the mammography recall scenario requiring non-invasive additional imaging, mean QOL increased from 55 during the period of diagnostic uncertainty to 68 after results were received. Similarly, mean QOL increased from 46 for a scenario in which biopsy was performed but results were not yet known, to 68 after benign results were delivered. In the MRI recall scenario requiring additional evaluation (Table E1), QOL increased from 47 during diagnostic uncertainty to 58 after results are received. In the MRI recall scenario requiring biopsy (Table E2), QOL increased from 44 prior to results being known to 62 after results are received. Of note, there was substantial within-woman variability in changes in QOL ratings before and after results were known, ranging from a decrease in QOL score by 52 to an improvement in QOL score of 95 across scenarios. Perceived risk was not a predictor of QOL scores in any of the scenarios.

Multivariable regression analysis indicated that across all screening recall scenarios, breast cancer worry was a significant predictor of decreased QOL during the period of diagnostic uncertainty while awaiting results from non-invasive additional imaging or biopsy (mean QOL scores ranged from 25 to 33 in women with high worry versus 45–57 in women without high worry). High trait anxiety (top quartile STAI percentile score) was a predictor of persistently lower QOL scores in one scenario of diagnostic uncertainty after MRI-guided biopsy (mean QOL scores 37 versus 45 in women with high versus low anxiety, respectively) and in all scenarios after receipt of benign results (mean QOL scores ranged from 50 to 60 in women with high anxiety versus 58–68 in women without high anxiety). Family history of breast cancer was also a predictor of persistently lower QOL scores after receipt of benign results in most (3 of 4) scenarios. Personal history of breast cancer and prior history of screening recall were not predictors of QOL ratings for any scenarios. Women who incorrectly answered one or more items on the numeracy measure had lower QOL ratings for screening mammography recall for additional imaging, but not for other scenarios. Age was a predictor of lower QOL score after MR-guided biopsy, but not in other scenarios. No other variables were predictors of QOL scores in multivariate analyses in any of the presented scenarios.

## 4 Discussion

Our vignette-based survey of women across a spectrum of risk undergoing breast cancer screening suggests that perceived QOL during recall after screening mammography or MRI does not appear to vary by breast cancer risk level. Across a spectrum of risk, QOL was lower during the period of diagnostic uncertainty, and then improved after benign results were received. Breast cancer worry was a predictor of lower QOL during the period of diagnostic uncertainty, and trait anxiety and family history were significant predictors of persistently low QOL after receipt of benign results.

Our finding that the perceived impact of screening recall does not differ between women at average and high risk of breast cancer may be explained by inaccurate breast cancer risk perceptions among the participants in our study. Interestingly, perceived breast cancer risk did not vary by actual risk category, with average and high-risk women estimating their lifetime risk of breast cancer to be 46% and 48%, respectively, and most (75%) average risk women incorrectly reporting >20% lifetime risk of breast cancer. These findings corroborate prior studies which have shown that women’s perceived risk of breast cancer is often inaccurate [[30], [31], [32], [33], [34]], although these studies have produced mixed results regarding whether women tend to overestimate their risk or underestimate their risk [32]. This discrepancy may in part be due to the different instruments used to assess perceived risk, as studies using numerical risk estimates (such as ours) suggest that women tend to overestimate their breast cancer risk [30,31,33], while studies using verbal scales suggest women tend to underestimate their risk [[33], [34], [35]].

Our findings are in keeping with the results of prior studies of women at average risk which have suggested that the period of diagnostic uncertainty is most distressing for women who have been recalled from screening, and that quality of life ratings improve on average after receipt of benign results [14]. However, the duration of psychosocial consequences in average risk women recalled from screening is unclear, as studies have demonstrated mixed results. While some studies have suggested that these effects are short-term[16,36], other studies have demonstrated evidence of persistent psychological distress for at least 12 months [11] to 36 months [14]. Interestingly, studies of high-risk women who have experienced screening recall suggest that breast cancer worry significantly decreases by 6 months after recall [36,37]. As breast cancer risk assessment improves, understanding the impact of screening recall by breast cancer risk level is important to informing risk-tailored breast cancer screening strategies. Our study is unique in that it included women at average, intermediate, and high risk for breast cancer, and our results suggest that across breast cancer risk levels, QOL scores improve on average after receipt of benign results.

In our study, trait anxiety was a predictor of persistently low QOL scores after receipt of benign results. In a prior study of Danish women recalled from screening mammography[38], trait anxiety predicted lower QOL ratings across all time points up to 12 months after screening. In particular, women with high trait anxiety who had a false positive result endorsed persistently lower QOL ratings than women with low trait anxiety and a breast cancer diagnosis. These findings suggest that trait anxiety may be an important characteristic to consider during individualized decision-making regarding breast cancer screening.

Our study has a few limitations worth noting. The participants in our study provided QOL ratings for hypothetical vignettes of the experience of screening recall for additional imaging and biopsy, and their perceptions may differ from women experiencing actual screening recall episodes. In addition, because of the nature of the scenario we could not assess short versus long-term consequences of screening recall. Finally, the women in our study received screening at a single institution and were predominantly non-Hispanic white women and highly educated, limiting generalizability.

In conclusion, our vignette-based survey study suggests breast cancer risk affects QOL during breast cancer screening recall less than other factors such as breast cancer worry and trait anxiety. Across the spectrum of risk, breast cancer worry predicted lower QOL ratings for recall during the period of diagnostic uncertainty while awaiting test results. While quality of life generally improved after receipt of benign results, women with high trait anxiety levels reported persistently decreased quality of life even after results were known. Our results suggest that these women may particularly benefit from discussions regarding the potential for false-positive test results when choosing a breast cancer screening regimen.

## Ethical approval

This study was HIPAA-compliant and approved by the Institutional Review Board of Massachusetts General Hospital.

## Declaration of competing interest

JML and KPL receive research support from GE Healthcare. GSG was previously a consultant for GE Healthcare until 2018.

## Figures and Tables

**Table E1:** Quality-of-life (QOL) scores during and after recall from screening magnetic resonance imaging (MRI) for additional ultrasound evaluation.

Characteristic	During Diagnostic Uncertainty After MRI Recall			After Benign Results Received After MRI Recall		
	Mean QOL Score (SD)	Univariate p value	Multivariable p value	Mean QOL Score (SD)	Univariate p value	Multivariable p value
Age	46.4 (27.0)	0.26		57.1 (27.2)	0.80	
Race					0.50	
White	46.1 (26.7)			57.1 (25.1)		
Nonwhite	60.2 (39.1)			64.8 (33.3)		
Numeracy Questions		0.08			0.22	
Both correct	48.8 (27.0)			58.7 (25.0)		
1+ incorrect	40.8 (26.4)			53.3 (25.8)		
Family History of BC^		0.91			0.13	
Yes	47.0 (27.5)			54.4 (26.1)		
No	45.9 (26.6)			59.7 (24.4)		
Personal History of BC^		0.91			0.90	
Yes	45.3 (29.5)			57.8 (25.9)		
No	45.9 (26.5)			57.2 (25.4)		
Risk		0.088			0.94	
Low/Intermediate	43.9 (27.1)			57.2 (24.4)		
High	51.6 (26.4)			56.9 (27.2)		
Perceived BC Risk (%)	46.9 (27.1)	0.52		57.2 (24.9)	0.69	
Anxiety Level		0.10			**0.04**	**0.0382**
Top quartile	40.9 (26.1)			50.5 (24.8)		
Bottom 3 quartiles	48.6 (27.1)			59.6 (25.1)		
BC worry∗		**0.002**	**0.004**		0.20	
High worry	27.6 (25.37)			49.6 (23.8)		
Low-moderate worry	48.6 (26.40)			58.0 (25.4)		

BC=Breast Cancer.

∗High worry was defined as a score ≥6 on the Breast Cancer Worry scale.

**Table E2:** Quality-of-life (QOL) scores during and after recall from screening MRI with MR-guided biopsy performed.

Characteristic	During Diagnostic Uncertainty After Biopsy			After Benign Results Received After Biopsy		
	Mean QOL Score (SD)	Univariate p value	Multivariable p value	Mean QOL Score (SD)	Univariate p value	Multivariable p value
Age	43.2 (27.5)	0.28		60.8 (26.7)	0.20	**0.008**
Race		0.09			0.27	
White	42.5 (27.3)			60.7 (26.7)		
Nonwhite	64.0 (32.3)			74.0 (23.8)		
Numeracy Questions		0.30			0.46	
Both correct	44.6 (27.2)			59.8 (26.1)		
1+ incorrect	39.7 (28.1)			63.2 (28.0)		
Family History of BC		0.50			**0.02**	**0.02**
Yes	41.9 (28.1)			55.8 (28.1)		
No	44.4 (26.9)			65.8 (24.3)		
Personal History of BC		0.48			0.41	
Yes	41.1 (29.4)			59.5 (27.5)		
No	44.9 (26.6)			63.7 (27.1)		
Risk		0.17			0.94	
Low/Intermediate	41.1 (27.0)			61.0 (25.5)		
High	47.4 (28.3)			60.6 (29.2)		
Perceived BC Risk (%)	42.8 (27.5)	0.75		59.4 (26.5)	0.06	
Anxiety Level		0.10	**0.04**		**0.04**	**0.04**
Top quartile	37.4 (25.4)			54.0 (25.0)		
Bottom 3 quartiles	45.4 (28.0)			64.0 (27.0)		
BC worry∗		**0.003**	**0.009**		0.34	
High worry	24.8 (23.4)			54.9 (24.3)		
Low-moderate worry	45.3 (27.2)			61.5 (26.9)		

BC=Breast Cancer.

∗High worry was defined as a score ≥6 on the Breast Cancer Worry scale.
